# Early diagnosis of autism and other developmental disorders, Brazil, 2013–2019

**DOI:** 10.11606/s1518-8787.2023057004710

**Published:** 2023-03-29

**Authors:** Vania Reis Girianelli, Jeane Tomazelli, Cosme Marcelo Furtado Passos da Silva, Conceição Santos Fernandes

**Affiliations:** I Fundação Oswaldo Cruz Escola Nacional de Saúde Pública Sérgio Arouca Departamento de Direitos Humanos, Saúde e Diversidade Cultural Rio de Janeiro RJ Brasil Fundação Oswaldo Cruz. Escola Nacional de Saúde Pública Sérgio Arouca. Departamento de Direitos Humanos, Saúde e Diversidade Cultural. Rio de Janeiro, RJ, Brasil; II Instituto Nacional de Câncer José Alencar Gomes da Silva Rio de Janeiro RJ Brasil Instituto Nacional de Câncer José Alencar Gomes da Silva. Rio de Janeiro, RJ, Brasil; III Fundação Oswaldo Cruz Escola Nacional de Saúde Pública Sérgio Arouca Departamento de Epidemiologia e Métodos Quantitativos em Saúde Rio de Janeiro RJ Brasil Fundação Oswaldo Cruz. Escola Nacional de Saúde Pública Sérgio Arouca. Departamento de Epidemiologia e Métodos Quantitativos em Saúde. Rio de Janeiro, RJ, Brasil; IV Pontifícia Universidade Católica do Rio de Janeiro Rio de Janeiro RJ Brasil Pontifícia Universidade Católica do Rio de Janeiro. Rio de Janeiro, RJ, Brasil

**Keywords:** Autism Spectrum Disorder, Epidemiology, Child Development Disorders, Pervasive, Early Diagnosis, Analytical Epidemiology, Psychosocial Support Systems, Unified Health System

## Abstract

**OBJECTIVE:**

To investigate the factors associated with the early diagnosis of autism and other types of pervasive developmental disorder (PDD) in children treated at the Psychosocial Care Center for Children and Adolescents of the Unified Health System, from 2013 to 2019,in Brazil.

**METHODS:**

An exploratory cross-sectional study, based on data from the Record of Outpatient Health Actions (RAAS) of the first appointment of children aged 1 to 12 years. The gross (RR_g_) and adjusted (RR_a)_ relative risks and respective 95% confidence intervals (95%CI) were estimated using the Poisson regression model with robust variance estimation.

**RESULTS:**

Of the 22,483 children included in the study, the majority were male (81.9%), lived in the same municipality where they were diagnosed (96.8%) and in the Southeast region (57.7%). Early diagnosis was higher for childhood autism (RR_g_ = 1.48; 95%CI 1.27–1.71) , PDD without subtype designation (RR_g_ = 1.55; 95%CI 1.34–1.80), other PDD (RR_g_ = 1.48; 95%CI 1.21–1.81) and PDD not otherwise specified (RR_g_ = 1.44; 95%CI 1.22–1.69) than for atypical autism. Children residing in the same municipality where the diagnosis was made had a higher rate of early diagnosis (RR_g_ = 1.31; 95%CI 1.10–1.55) than the others; as well as those referred by primary care (RR_g_ = 1.51; 95%CI 1.37–1.68) and by spontaneous demand (RR_g_ = 1.45; 95%CI 1.31–1.61) than those from other types of referral. Early diagnosis was higher from 2014 and lower in the North region than in the other regions. In the multiple analysis, the magnitude of RR_a_ was similar to that of RR_g_.

**CONCLUSIONS:**

Early identification of autism and other PDD has improved in Brazil, but it still represents about 30% of the diagnoses made. The variables included in the model were significant, but still explain little of the early diagnosis of children with autism and other PDD.

## INTRODUCTION

Autism is a neurodevelopmental disorder and, in Brazil, the cases seen in the Unified Health System (SUS) are part of a diagnostic category specified as pervasive developmental disorders (PDD), according to the 10th International Statistical Classification of Diseases and Related Health Problems (ICD-10). The pervasive disorder, unlike a specific disorder, affects a variety of psychic functions and is also called invasive developmental disorder^1^. This diagnostic category includes some neurological disorders with a great impact on child neurodevelopment, with symptoms mainly of social interaction and communication deficits and repetitive and restrictive behaviors^2^. In the new version of the classification (ICD-11), to be implemented in 2022, this category is now called autism spectrum disorder (ASD) and excludes Rett syndrome and disorder with hyperkinesia and retardation, closer to the 5th revision of the Diagnostic and Statistical Manual of Mental Disorders (DSM-5) and emphasizing cognition, intellectual capacity and functional language^3 , 4^.

Children with ASD tend to have developmental problems between 12 and 24 months^5 , 6^, but the warning signs can be perceived before they reach one year^7 , 8^. Several authors bring converging data that early diagnosis favors and enhances the possibilities of intervention in the early stages of child development by enabling the acquisition of repertoire, such as the development of cognitive skills, like verbal language and communication^9^; sociocognitive skills, such as shared attention^10^; and behavioral skills, such as autonomy and social skills^11^. Some authors also describe that early diagnosis helps to better guide parents through psychoeducation and the development of management strategies^12^. In this sense, the importance of early diagnosis of autism is increasingly evident in the literature, due to the potential impact of the intervention, which provides stimulation for the child. This is because, in the first years of life, there is a greater capacity for neural organization, which favors a better prognosis and quality of life^13^.

Parents are usually the first to suspect^5 , 6^, but lack of knowledge about aspects of development expected for each age can delay the search for assistance. The primary health care professional is the population’s first contact in the health network and must be aware of the atypical development for appropriate referral of suspected cases^14^. In the SUS, care is carried out in the Psychosocial Care Network (RAPS), and the Psychosocial Care Center for Children and Adolescents (CAPSi) is the specialized center for toddler and teenagers. The CAPSi must be articulated with other points in the network, such as the Basic Health Units (UBS) and the Specialized Rehabilitation Centers (CER), and integrated into the Care Network for Persons with Disabilities, ensuring comprehensive care^1 , 15^. Despite the population parameter for enabling a CAPSi^15^ (from 70,000 inhabitants), Tomazelli et al.^16^identified 246 CAPSi in the country distributed in 206 municipalities, which reported serving children up to 12 years of age with PDD, of which 33,7% had a population between 70 and 199 thousand inhabitants and 48.8% had more than 200 thousand inhabitants.

Thus, the objective of this study is to investigate the factors associated with the early diagnosis of autism and some types of pervasive developmental disorder in children treated at the Psychosocial Care Center for Children and Adolescents of the Unified Health System, from 2013 to 2019, in Brazil.

## METHODS

This is an exploratory cross-sectional study on the factors associated with the early diagnosis of children with autism and other PDD diagnoses. The data were accessed on the RAAS, and the diagnosis reported on the platform uses ICD-10. The extraction and selection process of the eligible population is available in a previous study^16^, in which 23,657 children up to 12 years of age were identified whose first appointment took place between 2013 and 2019.

**Figure 1:**
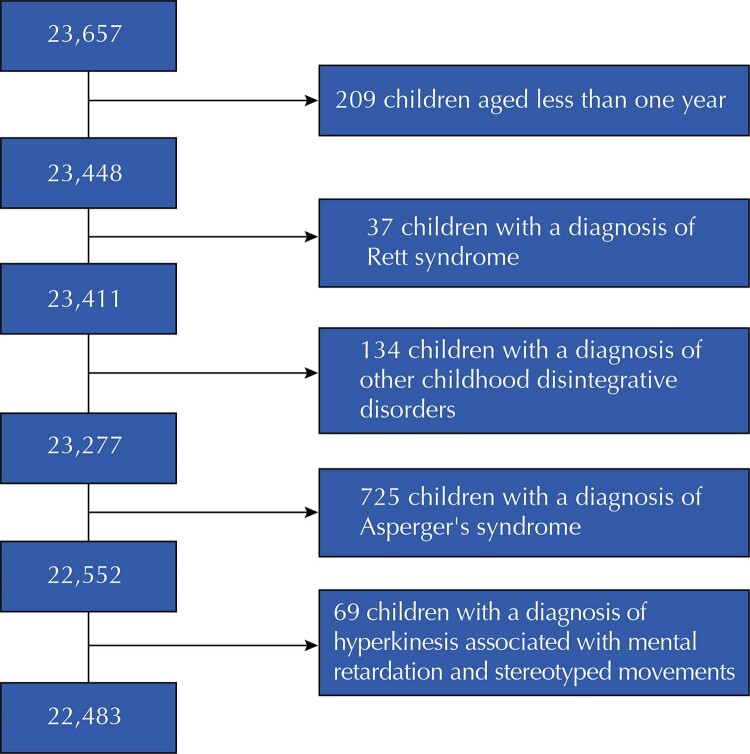
Identification of eligible population for the study.

From this database, 1,174 children were excluded: 209 aged less than 1 year, 37 with an initial diagnosis of Rett syndrome (F84.2), 134 with other childhood disintegrative disorders (F84.3), 725 with Asperger’s syndrome (F84.5) and 69 with disorders with hyperkinesia associated with mental retardation and stereotyped movements (F84.4) ( Figure 1 ). Children younger than 1 year old were excluded due to the fragility of diagnoses, since at this stage it is possible to identify signs of risk and vulnerability through indications of interaction and communication disorders^1^, or due to the limitation of recording the diagnostic suspicion in the information system^16^. The other exclusions were carried out considering that: Rett syndrome and disorders with hyperkinesia associated with mental retardation and stereotyped movements will form another diagnostic category in ICD-11 and will therefore not be part of the ASD diagnostic category^3^; children with childhood disintegrative disorders have normal motor and intellectual development up to 3 years of age or older and only later begin to lose acquired capacities; and children diagnosed with Asperger have preserved language and a good cognitive level^1^.

PDD without specification of type (F84) and diagnoses of childhood autism (F84.0), atypical autism (F84.1), other PDD (F84.8) and PDD not otherwise specified (F84.9) were included in the study. In the end, the database had 22,483 records of the first appointment of children between 1 and 12 years of age.

The outcome of interest is early diagnosis, which was defined as a diagnosis made before 4 years of age, and other diagnoses considered late. The explanatory variables evaluated regarding the eligible population were: gender, race/skin color, macro-region of residence, patient’s origin (spontaneous demand, primary care, emergency service, other Psychosocial Care Centers, day hospital and psychiatric hospital), initial diagnosis, year of diagnosis, and whether the municipality of residence was the same as that of diagnosis. The municipal human development index (HDI) was also evaluated for the child’s residence for the year 2010, categorized into: very low (0.000 to 0.499), low (0.500 to 0.599), moderate (0.600 to 0.699), high (0.700 to 0.799) and very high (0.800 to 1.00)^17^.

Measures of central tendency (mean and median) and dispersion (amplitude, standard deviation, and 1st and 3rd quartiles) were calculated for age, type of diagnosis and region; as well as the percentage of each category of the other variables studied, stratified by outcome. Pearson’s chi-square test was used to assess the existence of a statistically significant difference (p ≤ 0.05) between strata of the group with early versus late diagnosis for the studied variables. To assess trends in the proportion of cases per year of diagnosis, the chi-square test for trends was applied^18^.

Data refer only to new cases identified between 2013 and 2019. Therefore, in order to investigate the variables associated with early diagnosis, the gross relative risk (RR_g_) was estimated in the bivariate analysis and, in the multiple analysis, the adjusted relative risk (RR_a_) for the other variables, using the Poisson regression model with robust variance estimation^19^. The statistical significance of each explanatory variable present in the model was assessed using the Wald test. In the multiple analysis, the initial model consisted of including explanatory variables with p < 0.20 in the bivariate analysis, with variables with a statistical significance level ≤ 0.05^20^ remaining in the final model. The explanatory capacity of the model was estimated considering the reduced percentage of the deviance function^21^.

Additionally, residual analysis was performed to assess the relative influence of observations on model fit. This evaluation was performed using a dot plot with standardized Pearson residuals versus leverage distance to identify outliers and their influence on the model.

Cook’s distance was also calculated, which combines the standardized residual with the leverage measure. When observations are outside the 0.5–1 range, it indicates the absence of outliers and/or influential dots that interfere with the observed association estimates^22^.

Data were analyzed in the statistical program R version 4.0.4, using the packages: *dplyr, psych, car, MASS, DescTools, QuantPsyc* and *ggplot2* .

## RESULTS

Of the 22,483 children included in the study, the majority were male (81.9%), lived in the Southeast region (57.7%) and in the same municipality where they were diagnosed (96.8%). A total of 6,835 (30.4%) children received early diagnosis ( Table 1 ). There was no subtype designation in the information system in 56.4% of the diagnoses, which were identified only as PDD. Next, the most frequent diagnoses were childhood autism (31.9%) and PDD not otherwise specified (7.0%). Only the sex variable did not present a statistically significant difference (p = 0.502). The analysis of the race/skin color variable was not feasible due to the high percentage of incompleteness (39.3%), but the analysis restricted to the white, black, brown and yellow categories showed a statistically significant difference. The year 2013 had the highest proportion of diagnoses in the period (18.9%); but from 2014 to 2018 the proportion increased progressively, with a reduction in 2019. Regarding early diagnosis, it ranged from 23.3% in 2013 to 32.8% in 2019, with a peak in 2017 (33.4%) and 2018 (34.9%), with a statistically significant linear trend (p < 0.001).

**Table 1:** Proportional distribution (%) of characteristics of children diagnosed with autism and other types of PDD treated at CAPSi according to early or late diagnosis. Brazil, 2013 to 2019.

Characteristics	Total		Diagnosis				

			Early		Late		p^b^
		
	n	%	n	%	n	%	
Sex							
Female	4,075	18.1	1,221	17.9	2,854	18.2	0.502
Male	18,408	81.9	5,614	82.1	12,794	81.8	
Race/color							
White	5,835	26.0	1,880	27.5	3,955	25.3	< 0.001
Black	461	2.1	107	1.6	354	2.3	
Brown	6,638	29.5	2,032	29.7	4,606	29.4	
Yellow	710	3.2	111	1.6	599	3.8	
Indigenous	5	0.0	2	0.0	3	0.0	-
Unknown	8,834	39.3	2,703	39.5	6,131	39.2	-
Origin							
Spontaneous demand	11,468	51.0	3,483	51.0	7,985	51.0	< 0.001
Primary care	9,719	43.2	3,081	45.1	6,638	42.4	
Other^a^	1,296	5.8	271	4.0	1,025	6.6	
Region of residence							
North	1,358	6.0	248	3.6	1,110	7.1	< 0.001
Northeast	5,495	24.4	1,867	27.3	3,628	23.2	
Midwest	897	4.0	249	3.6	648	4.1	
Southeast	12,969	57.7	4,053	59.3	8,916	57.0	
South	1,764	7.8	418	6.1	1,346	8.6	
Municipality of residence the same as municipality of diagnosis							
Yes	21,773	96.8	6,656	97.4	15,117	96.6	0.002
No	710	3.2	179	2.6	531	3.4	
Initial diagnosis (ICD)							
General PDD (F84)	12,670	56.4	3,978	58.2	8,692	55.5	< 0.001
Childhood autism (F84.0)	7,177	31.9	2,143	31.4	5,034	32.2	
Atypical Autism (F84.1)	628	2.8	127	1.9	501	3.2	
Other PDD (F84.8)	445	2.0	133	1.9	312	2.0	
PDD not otherwise specified (F84.9)	1,563	7.0	454	6.6	1,109	7.1	
Year of diagnosis							
2013	4,242	18.9	989	14.5	3,253	20.8	< 0.001
2014	2,490	11.1	723	10.6	1,767	11.3	
2015	2,669	11.9	782	11.4	1,887	12.1	
2016	2,932	13.0	914	13.4	2,018	12.9	
2017	3,529	15.7	1,179	17.2	2,350	15.0	
2018	3,645	16.2	1,271	18.6	2,374	15.2	
2019	2,976	13.2	977	14.3	1,999	12.8	
Municipal HDI							
Very low	761	3.4	232	3.4	529	3.4	0.008
Low	4,378	19.5	1,424	20.8	2,954	18.9	
Moderate	9,923	44.1	2,970	43.5	6,953	44.4	
High	7,421	33.0	2,209	32.3	5,212	33.3	

^a ^Other: emergency service, other Psychosocial Care Centers, day hospital and psychiatric hospital.

^b ^p value of Pearson’s chi-squared test.

Among the diagnoses addressed, Brazil presented, in this period, a proportion of childhood autism diagnosis of 31.9% in the CAPSi, ranging from 23.7% in the North to 39.0% in the Northeast. PDD without classification had a higher proportion in the North (73.1%) and lower in the South (36.5%), the region that had, however, the highest proportion of PDD not otherwise specified (17.5%). Mean and median age were higher for atypical autism, while median age was the same for other disorders. Regarding the regions, some variations were observed: the North region had a higher mean age for atypical autism and a lower age for other PDD – however with few cases, and the first quartile higher than the distribution in Brazil for all diagnoses. For the Northeast and Southeast regions, as well as for Brazil, with the exception of the diagnosis of atypical autism, early diagnosis occurred in 25% of cases ( Table 2 ).

**Table 2:** Proportional distribution of the diagnosis of autism and other types of PDD treated at the CAPSi and respective mean and median age at diagnosis according to region of residence. Brazil, 2013 to 2019.

Region of residence	Diagnosis	n	%	Age of child (years)						

				Mean	Standard deviation	Median	1st Quartile	3rd Quartile	Min.	Max.
North	Atypical autism	13	1.0	7.3	2.0	6.0	6.0	9.0	5	11
	Childhood autism	322	23.7	6.3	2.7	6.0	4.0	8.0	1	12
	General PDD	993	73.1	6.1	2.8	6.0	4.0	8.0	1	12
	Other PDD	2	0.1	4.5	0.7	4.5	4.3	4.8	4	5
	PDD not otherwise specified	28	2.1	5.8	2.7	5.5	3.8	7.0	2	12
	Subtotal	1,358	100.0	6.2	2.7	6.0	4.0	8.0	1	12
Northeast	Atypical autism	203	3.7	5.9	2.8	5.0	4.0	7.5	1	12
	Childhood autism	2,144	39.0	5.0	2.7	4.0	3.0	7.0	1	12
	General PDD	2,798	50.9	5.2	2.8	5.0	3.0	7.0	1	12
	Other PDD	133	2.4	5.3	2.9	5.0	3.0	7.0	1	12
	PDD not otherwise specified	217	3.9	5.4	2.6	5.0	3.0	7.0	1	12
	Subtotal	5,495	100.0	5.2	2.8	4.0	3.0	7.0	1	12
Midwest	Atypical autism	36	4.0	6.3	2.8	5.5	4.0	9.0	2	12
	Childhood autism	235	26.2	5.6	2.8	5.0	3.0	8.0	1	12
	General PDD	590	65.8	5.7	2.9	5.0	3.0	8.0	1	12
	Other PDD	6	0.7	6.7	4.3	5.0	3.3	10.5	3	12
	PDD not otherwise specified	30	3.3	5.9	2.9	6.0	4.0	8.0	1	12
	Subtotal	897	100.0	5.7	2.8	5.0	3.0	8.0	1	12
Southeast	Atypical autism	308	2.4	6.3	3.0	6.0	4.0	9.0	1	12
	Childhood autism	3,815	29.4	5.5	2.8	5.0	3.0	7.0	1	12
	General PDD	7,646	59.0	5.4	2.9	5.0	3.0	7.0	1	12
	Other PDD	220	1.7	6.0	3.1	6.0	3.0	8.0	1	12
	PDD not otherwise specified	980	7.6	5.6	2.9	5.0	3.0	7.0	1	12
	Subtotal	12,969	100.0	5.5	2.9	5.0	3.0	7.0	1	12
South	Atypical autism	68	3.9	6.2	3.0	6.0	4.0	9.0	1	12
	Childhood autism	661	37.5	6.3	2.9	6.0	4.0	8.0	1	12
	General PDD	643	36.5	6.1	3.1	6.0	4.0	8.0	1	12
	Other PDD	84	4.8	5.8	2.9	5.0	4.0	8.0	1	12
	PDD not otherwise specified	308	17.5	5.6	3.1	5.0	3.0	8.0	1	12
	Subtotal	1,764	100.0	6.1	3.0	6.0	4.0	8.0	1	12
Brazil	Atypical autism	628	2.8	6.2	2.9	6.0	4.0	9.0	1	12
	Childhood autism	7,177	31.9	5.5	2.8	5.0	3.0	7.0	1	12
	General PDD	12,670	56.4	5.4	2.9	5.0	3.0	7.0	1	12
	Other PDD	445	2.0	5.8	3.0	5.0	3.0	8.0	1	12
	PDD not otherwise specified	1,563	7.0	5.6	2.9	5.0	3.0	7.0	1	12
	Subtotal	22,483	100.0	5.5	2.9	5.0	3.0	7.0	1	12

PDD: pervasive developmental disorder.

Table 3 presents the results of RR_g_ and RR_a_ and the respective 95% confidence intervals (95%CI). Early diagnosis of children with childhood autism was 48% higher than that of atypical autism. The other categories also had a higher early diagnosis than atypical autism: PDD without subtype designation (RR_g_ = 1.55), other PDD (RR_g_ = 1.48) and PDD not otherwise specified (RR_g_ = 1.44).

**Table 3:** Gross and adjusted relative risk of factors associated with early diagnosis of children diagnosed with autism and other types of PDD treated at CAPSi. Brazil, 2013 to 2019.

Variables	RRg	95%CI	RRa	95%CI
Initial diagnosis				
Atypical autism	1.00		1.00	-
Childhood autism	**1.48**	1.27–1.71	**1.43**	1.23–1.66
General PDD	**1.55**	1.34–1.80	**1.50**	1.29–1.74
Other PDD	**1.48**	1.21–1.81	**1.49**	1.21–1.83
PDD not otherwise specified	**1.44**	1.22–1.69	**1.49**	1.26–1.76
Origin				
Other^a^	1.00		1.00	
Primary care	**1.51**	1.37–1.68	**1.40**	1.26–1.55
Spontaneous demand	**1.45**	1.31–1.61	**1.29**	1.16–1.43
Year of diagnosis				
2013	1.00	-	1.00	-
2014	**1.25**	1.15–1.35	**1.25**	1.15–1.35
2015	**1.26**	1.16–1.36	**1.24**	1.15–1.35
2016	**1.34**	1.24–1.44	**1.33**	1.23–1.44
2017	**1.43**	1.34–1.54	**1.41**	1.37–1.58
2018	**1.50**	1.40–1.60	**1.47**	1.37–1.58
2019	**1.40**	1.31–1.52	**1.40**	1.30–1.50
Region of residence				
North	1.00	-	1.00	-
Northeast	**1.86**	1.67–2.08	**1.90**	1.70–2.13
Midwest	**1.52**	1.31–1.76	**1.51**	1.31–1.76
Southeast	**1.71**	1.54–1.90	**1.69**	1.52–1.88
South	**1.29**	1.14– 1.48	**1.27**	1.11– 1.45
Municipality of residence the same as municipality of diagnosis				
No	1.00	-	1.00	
Yes	**1.31**	1.10–1.55	**1.27**	1.11–1.42
Municipal HDI^b^				
Very low	1.00	-		
Low	1.07	0.95–1.20		
Moderate	0.98	0.88–1.10		
High	0.98	0.87–1.09		
Sex				
Female	1.00	-		
Male	1.02	0.97–1.07		

^a ^Other: emergency service, other Psychosocial Care Centers, day hospital and psychiatric hospital.

^b ^p value in multiple analysis > 0.05.

Children referred by primary care and those whose origin was spontaneous demand received more early diagnoses, respectively 51% and 45%, than those from other types of referral. Children residing in the same municipality where they received the diagnosis had 31% more early diagnoses than the others. Early diagnosis was higher from 2014 and lower in the North region as compared to the other regions.

In the multiple analysis, the only variable that was not included in the model was sex, as it was not statistically significant in the bivariate analysis (p > 0.20). The municipal HDI variable was the only one that was not statistically significant in the multiple analysis (p > 0.05) and, therefore, was not maintained in the model. Of the variables included in the model for the multiple analysis, there was no expressive change in the magnitude of the association, that is, the independent effect of each variable after being adjusted by the others was similar to the unadjusted one (gross). The model showed, however, a low predictive capacity (2.0%) and an inadequate overall fit (p < 0.001).

**Figure 2:**
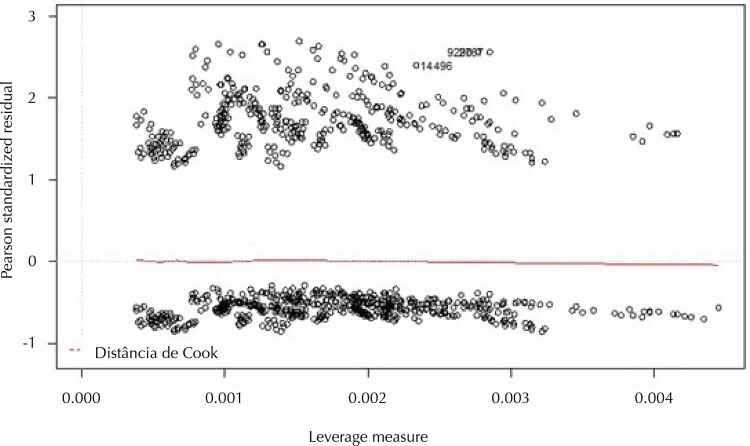
Pearson standardized residuals versus leverage measure and respective Cook’s distance of the regression model for the early diagnosis of children diagnosed with autism and other types of PDD treated at CAPSi. Brazil, 2013 to 2019.

Figure 2 presents the residual analysis that indicates the absence of influential cases: the leverage measure ranged from > 0.000 to < 0.005, Pearson’s standardized residuals from 0.79 to 2.88, and no observation was within the Cook’s distance cut-off point.

## DISCUSSION

The study identified an early and increasing diagnosis over the years studied, with a statistically significant linear trend. The proportion of diagnoses in the period, however, was higher in 2013 and lower in 2019. As in 2013 the services performed at CAPSi began to be recorded in RAAS^23^, it is plausible to assume that the change in the recording instrument, previously performed in Authorization for High Complexity Procedures, may have generated an accumulation of records, which were released in that first year. The reduction in 2019 may express a real drop, requiring an evaluation with a longer series of years.

In a study that evaluated 126 Brazilian autistic children living in 19 states, the age at diagnosis ranged from 16 to 204 months, with most parents having higher education (63.3%) and high socioeconomic status (76.4%)^24^. The age at diagnosis was weakly correlated with the age of recognition of the first signs of ASD and was not statistically significantly correlated with the family’ socioeconomic level, nor with the child’s birth order and the signs of autism. The authors indicated, however, that it is possible that in families with lower socioeconomic status, the diagnosis of children with autism occurs later. They also highlighted that the mean age of diagnosis of autism in Brazil is advanced as compared to children diagnosed in other countries.

Late diagnosis, however, is still an international reality. The reasons may include pediatricians’ non-adherence to evaluation protocols, variation in experience, and the use of non-validated instruments^25^. In a monitoring study carried out in 11 cities in the United States, the children’s mean age at the time of diagnosis was 51 months, which may vary depending on the intelligence quotient (IQ) and race/skin color^26^. A review study that evaluated the influence of child, family and community characteristics on the age of diagnosis of ASD^27^found a variation of mean age from 38 to 120 months and of median age from 34 to 88 months for the entire spectrum and with a tendency to decrease over time. Therefore, the results indicated that the diagnosis of children with autism has occurred increasingly earlier. This study also identified that symptom severity and access to health and education systems were determining factors for early diagnosis and that the performance of local policies and infrastructure seem to cause differences in diagnostic age across regions. In this article, the mean age in Brazil, for the period studied, ranged from 5.4 years (64.8 months for general PDD) to 6.2 years (74.4 months for atypical autism), while the median age was 5 years (60 months), except for atypical autism, which was greater. The exclusion criteria for some PDD diagnoses used suggest that the mean age of the studied diagnoses, in children up to 12 years of age, may approach the mean age for diagnosis of ASD in the country, despite the fact that diagnoses of other childhood disintegrative disorders and Asperger were not maintained. Furthermore, the variation in the mean age found actually indicates a later diagnosis in Brazil; and that having limited the study population to children up to 12 years of age may have influenced the means found, since the diagnosis can occur at older ages.

In this study, the probability of early diagnosis differed across regions, being higher in the Northeast and Southeast than in the North, even as adjusted for the other variables included in the model. Differences between the Brazilian macro-regions in the distribution of various diagnoses of care provided at CAPSi were identified in a previous study^28^, which may affect the occurrence of autism and other PDD. Even so, a recent study found a growing trend in the early diagnosis of children with PDD^16^. Although the literature points to an association between parents’ higher educational level and early diagnosis^27^, the inclusion of the municipal HDI, as an indicator of the local educational level, was not significant. The results of this study also show differences in the means and medians of age at diagnosis across the regions, with higher means, in general, for the North and South regions, reiterating the later diagnosis.

Carrying out the diagnosis in the municipality of residence favors early identification of PDD, but most municipalities do not have CAPSi^16^, although it is assumed that the health care network is regionalized, with established care flows and guaranteed comprehensive care. Referral from primary care also contributed to early diagnosis, but the high association with spontaneous demand is noteworthy. A recent study addressed the relationship between the perception of stigma by caregivers of children with ASD and the difficulty in accessing health services in Latin America^29^. An important finding was that access barriers were more perceived by caregivers of female preschool children. Specifically in relation to Brazil, the results indicated that the perceptions of access barriers, frustration in accessing the service, the feeling of helplessness and the negative impact perceived by caregivers were greater. These results highlight the difficulty of accessing health services at the stage in which an early diagnosis should be made.

There is heterogeneity in the training and distribution of professionals in Brazil and the availability of multidisciplinary teams^2 , 30^, which may contribute to the variability of PDD diagnoses between regions, as identified by Ceballos et al.^28^and by this study. Most PDDs were not typified, but in the North this proportion was greater than 73%, while atypical autism represented 1%, levels that are very different from the national value. In addition, autism has a variety of symptoms, including associated comorbidities, and does not yet have established biological or environmental determinants that are associated with the manifestation of the disorder^2^, therefore, it is necessary to improve diagnostic routines, improve and qualify guidelines for these practices, as well as for support and intervention systems for children and their families .

Studies that include contextual variables, which, however, are still scarce, can contribute to the understanding of the factors associated with the occurrence of ASD. In this study, it was not possible to use the race/skin color variable due to incompleteness of the records and the fact that the database does not include variables that would allow a more comprehensive evaluation, limiting the analysis to the restricted number of variables available in the system, which probably compromised the predictive ability. A limitation of the study is the lack of information about the diagnostic methods used, which could further qualify the discussion. Enrichment of the database through the inclusion of other explanatory variables can help to adjust the model. Still, this study is unprecedented in using public RAAS data to assess variables that influence early diagnosis.

## CONCLUSION

Early diagnosis of autism and other PDD has improved in the country, but it still represents about 30% of the diagnoses made, most of which have not yet been typified, which can compromise the quality of care. The type of diagnosis, the origin of the referral, the place of residence and the year of diagnosis influenced its occurrence. The variables included in the model, although significant, explain little about early diagnosis. Advancing in studies that include environmental and contextual variables can help to broaden the understanding of the subject and contribute to minimize the impact of missed intervention opportunities, with a consequent improvement in this population’s quality of life.
