# Supplementary material for: Early diagnosis of autism and other developmental disorders, Brazil, 2013–2019
Source: Rev Saude Publica. 2023 Mar 29;57:21. doi: 10.11606/s1518-8787.2023057004710 (PMC10118402; doi:10.11606/s1518-8787.2023057004710)
Supplement: Translation

# Translation: Diagnóstico precoce do autismo e outros transtornos do desenvolvimento, Brasil, 2013–2019

**Authors:** Vania Reis Girianelli, Jeane Tomazelli, Cosme Marcelo Furtado Passos da Silva, Conceição Santos Fernandes

**Affiliations:** I Brasil Fundação Oswaldo Cruz. Escola Nacional de Saúde Pública Sérgio Arouca. Departamento de Direitos Humanos, Saúde e Diversidade Cultural. Rio de Janeiro, RJ, Brasil; II Brasil Instituto Nacional de Câncer José Alencar Gomes da Silva. Rio de Janeiro, RJ, Brasil; III Brasil Fundação Oswaldo Cruz. Escola Nacional de Saúde Pública Sérgio Arouca. Departamento de Epidemiologia e Métodos Quantitativos em Saúde. Rio de Janeiro, RJ, Brasil; IV Brasil Pontifícia Universidade Católica do Rio de Janeiro. Rio de Janeiro, RJ, Brasil

**Keywords:** Transtorno do Espectro Autista, Epidemiologia, Transtornos Globais do Desenvolvimento Infantil, Diagnóstico Precoce, Epidemiologia Analítica, Sistemas de Apoio Psicossocial, Sistema Único de Saúde

## Abstract

**OBJETIVO:**

Investigar os fatores associados ao diagnóstico precoce do autismo e de outros tipos de transtorno global do desenvolvimento (TGD) de crianças atendidas no Centro de Atenção Psicossocial Infantojuvenil do Sistema Único de Saúde, no período de 2013 a 2019, no Brasil.

**MÉTODOS:**

Estudo transversal exploratório, com base nos dados do Registro das Ações Ambulatoriais de Saúde (RAAS) do primeiro atendimento de crianças de 1 a 12 anos. Foram estimados o risco relativo bruto (RR_b_) e ajustado (RR_a_), e respectivo intervalo de confiança de 95% (IC95%), utilizando o modelo de regressão de Poisson com estimativa de variância robusta.

**RESULTADOS:**

Das 22.483 crianças incluídas no estudo, a maioria era do sexo masculino (81,9%), residia no mesmo município em que foi diagnosticada (96,8%) e na região Sudeste (57,7%). O diagnóstico precoce foi maior para autismo infantil (RR_b_= 1,48; IC95% 1,27–1,71), TGD sem designação de subtipo (RR_b_= 1,55; IC95% 1,34–1,80), outros TGD (RR_b_= 1,48; IC95% 1,21–1,81) e TGD não especificado (RR_b_= 1,44; IC95% 1,22–1,69) do que para autismo atípico. As crianças que residiam no mesmo município onde foi realizado o diagnóstico tiveram maior índice de diagnóstico precoce (RR_b_= 1,31; IC95% 1,10–1,55) do que as demais; bem como aquelas encaminhadas pela atenção básica (RR_b_= 1,51; IC95% 1,37–1,68) e por demanda espontânea (RR_b_= 1,45; IC95% 1,31–1,61) do que as oriundas de outros tipos de encaminhamento. O diagnóstico precoce foi maior a partir de 2014 e menor na região Norte quando comparada às demais. Na análise múltipla, a magnitude do RR_a_foi similar ao do RR_b_.

**CONCLUSÕES:**

A identificação precoce de autismo e outros TGD tem melhorado no país, mas ainda representa cerca de 30% dos diagnósticos realizados. As variáveis incluídas no modelo foram significativas, mas ainda explicam pouco do diagnóstico precoce de crianças com autismo e outros TGD.

## INTRODUÇÃO

O autismo é um transtorno do neurodesenvolvimento e, no Brasil, os casos atendidos no Sistema Único de Saúde (SUS) integram uma categoria diagnóstica especificada como transtornos globais do desenvolvimento (TGD), segundo a 10ª Classificação Estatística Internacional de Doenças e Problemas Relacionados à Saúde (CID-10). O transtorno global, diferente de um transtorno específico, afeta uma variedade de funções psíquicas e também é chamado de transtorno invasivo do desenvolvimento^1^. Essa categoria diagnóstica compreende alguns distúrbios neurológicos com grande impacto no neurodesenvolvimento infantil, apresentando como sintomas principalmente déficit de interação social e de comunicação e comportamentos repetitivos e restritivos^2^. Na nova versão da classificação (CID-11), a ser implantada em 2022, essa categoria passa a ser denominada transtorno do espectro autista (TEA) e exclui a síndrome de Rett e o transtorno com hipercinesia e retardo, aproximando-se da 5ª revisão do Manual Diagnóstico e Estatístico de Transtornos Mentais (DSM-5) e enfatizando cognição, capacidade intelectual e linguagem funcional^3 , 4^.

Crianças com TEA tendem a apresentar problemas no desenvolvimento entre os 12 e 24 meses^5 , 6^, mas os sinais de alerta podem ser percebidos antes de completarem um ano^7 , 8^. Diversos autores trazem dados convergentes de que o diagnóstico precoce favorece e potencializa as possibilidades de intervenção em fases iniciais do desenvolvimento infantil por possibilitar a aquisição de repertório, como o desenvolvimento das habilidades: cognitivas, como a linguagem verbal e comunicação^9^; sociocognitivas, como a atenção compartilhada^10^; e comportamentais, como autonomia e habilidades sociais^11^. Alguns autores também descrevem que o diagnóstico precoce auxilia na melhor orientação de pais através da psicoeducação e do desenvolvimento de estratégias de manejo^12^. Neste sentido, a importância do diagnóstico precoce do autismo fica cada vez mais evidente na literatura, em função do impacto potencial da intervenção, que propicia a estimulação da criança. Isso porque, nos primeiros anos de vida, há maior capacidade de organização neural, o que favorece melhor prognóstico e qualidade de vida^13^.

Os pais são, geralmente, os primeiros a suspeitarem^5 , 6^, mas o desconhecimento dos aspectos do desenvolvimento esperados para cada idade pode retardar a procura por assistência. O profissional da atenção primária em saúde é o primeiro contato da população na rede de saúde e deve estar atento ao desenvolvimento atípico para o encaminhamento adequado dos casos suspeitos^14^. No SUS, o atendimento é realizado na Rede de Atenção Psicossocial (RAPS), e o Centro de Atenção Psicossocial Infantojuvenil (CAPSi) é o centro especializado para crianças e adolescentes. O CAPSi deve estar articulado com outros pontos da rede, como as Unidades Básicas de Saúde (UBS) e os Centros Especializados em Reabilitação (CER), e integrada à Rede de Cuidados à Pessoa com Deficiência, assegurando a integralidade do cuidado^1 , 15^. Apesar do parâmetro populacional para a habilitação de um CAPSi^15^(a partir de 70 mil habitantes), Tomazelli e colaboradores^16^identificaram no país 246 CAPSi distribuídos em 206 municípios, que informaram atender crianças até 12 anos de idade com TGD, dos quais 33,7% apresentavam população entre 70 e 199 mil habitantes e 48,8% com mais de 200 mil habitantes.

Assim, o objetivo deste estudo é investigar os fatores associados ao diagnóstico precoce do autismo e de alguns tipos de transtorno global do desenvolvimento de crianças atendidas no Centro de Atenção Psicossocial Infantojuvenil do Sistema Único de Saúde, no período 2013 a 2019, no Brasil.

## MÉTODOS

Trata-se de estudo transversal exploratório sobre os fatores associados ao diagnóstico precoce de crianças com autismo e outros diagnósticos de TGD. Os dados foram acessados no RAAS, e o diagnóstico informado na plataforma utiliza a CID-10. O processo de extração e seleção da população elegível está disponível em estudo prévio^16^, em que foram identificadas 23.657 crianças até 12 anos de idade cujo primeiro atendimento ocorreu no período de 2013 a 2019.

Dessa base de dados foram excluídas 1.174 crianças, sendo 209 com menos de 1 ano, 37 com diagnóstico inicial de síndrome de Rett (F84.2), 134 com outros transtornos desintegrativos da infância (F84.3), 725 com síndrome de Asperger (F84.5) e 69 com transtornos com hipercinesia associada a retardo mental e a movimentos estereotipados (F84.4) ( Figura 1 ). Crianças menores de 1 ano foram excluídas pela fragilidade de diagnósticos, visto que nessa fase é possível identificar sinais de risco e vulnerabilidade através de indicativos de transtornos de interação e comunicação^1^, ou pela limitação de registrar a suspeita diagnóstica no sistema de informação^16^. As demais exclusões foram realizadas considerando que: a síndrome de Rett e os transtornos com hipercinesia associados a retardo mental e a movimentos estereotipados passarão a compor outra categoria diagnóstica na CID-11 e não farão parte, portanto, da categoria diagnóstica de TEA^3^; crianças com transtornos desintegrativos da infância apresentam desenvolvimento motor e intelectual normal até os 3 anos ou mais e só posteriormente começam a perder as capacidades adquiridas; e crianças com diagnóstico de Asperger apresentam preservação da linguagem e bom nível cognitivo^1^.

**Figura 1:** Identificação da população elegível para o estudo.

Foram incluídos no estudo o TGD sem especificação do tipo (F84) e os diagnósticos de autismo infantil (F84.0), autismo atípico (F84.1), outros TGD (F84.8) e TGD não especificado (F84.9). A base de dados ficou, ao final, com 22.483 registros do primeiro atendimento de crianças de 1 a 12 anos de idade.

O desfecho de interesse é o diagnóstico precoce, que foi definido como diagnóstico realizado antes dos 4 anos de idade, e os demais diagnósticos considerados tardios. As variáveis explicativas avaliadas referentes à população elegível foram: sexo, raça/cor da pele, macrorregião de residência, origem do paciente (demanda espontânea, atenção básica, serviço de urgência, outros Centros de Atenção Psicossocial, hospital dia e hospital psiquiátrico), diagnóstico inicial, ano do diagnóstico, e se o município de residência era igual ao de diagnóstico. Também foi avaliado o índice de desenvolvimento humano municipal (IDHM) referente à residência da criança para o ano de 2010, categorizado em: muito baixo (0,000 a 0,499), baixo (0,500 a 0,599), moderado (0,600 a 0,699), alto (0,700 a 0,799) e muito alto (0,800 a 1,00)^17^.

Foram calculadas as medidas de tendência central (média e mediana) e de dispersão (amplitude, desvio padrão, e 1º e 3º quartis) para idade, tipo de diagnóstico e região; bem como o percentual de cada categoria das demais variáveis estudadas, estratificado pelo desfecho. Utilizou-se o teste qui-quadrado de Pearson para avaliar a existência de diferença estatisticamente significativa (p ≤ 0,05) entre os estratos do grupo com diagnóstico precoce comparado ao tardio para as variáveis estudadas. Para avaliar a tendência na proporção de casos por ano do diagnóstico, aplicou-se o teste qui-quadrado para tendência^18^.

Os dados referem-se apenas aos casos novos identificados entre os anos de 2013 e 2019. Diante disso, para investigar as variáveis associadas ao diagnóstico precoce foram estimados, na análise bivariada, o risco relativo bruto (RR_b_) e, na análise múltipla, o risco relativo ajustado (RR_a_) para as demais variáveis, utilizando o modelo de regressão de Poisson com estimativa de variância robusta^19^. A significância estatística de cada variável explicativa presente no modelo foi avaliada por meio do teste de Wald. Na análise múltipla, o modelo inicial consistiu na inclusão das variáveis explicativas com p < 0,20 na análise bivariada, permanecendo no modelo final as variáveis com nível de significância estatística ≤ 0,05^20^. A capacidade explicativa do modelo foi estimada considerando a percentagem da função desvio ( *deviance* ) reduzida^21^.

Adicionalmente, realizou-se a análise de resíduo para avaliar a influência relativa das observações no ajuste do modelo. Essa avaliação foi realizada utilizando gráfico de pontos com resíduos padronizados de Pearson versus alavancagem (distância de *leverage* ) para identificação de casos atípicos e sua influência no modelo.

Também calculou-se a distância de Cook, que combina o resíduo padronizado com a medida de alavancagem. Quando as observações estão fora do intervalo de 0,5 a 1, indica a ausência de pontos atípicos e/ou influentes que interferem nas estimativas de associação observadas^22^.

Os dados foram analisados no programa estatístico R versão 4.0.4, utilizando os pacotes: *dplyr, psych, car, MASS, DescTools, QuantPsyc* e *ggplot2* .

## RESULTADOS

Das 22.483 crianças incluídas no estudo, a maioria era do sexo masculino (81,9%), residia na região Sudeste (57,7%) e no mesmo município em que foi diagnosticada (96,8%). Um total de 6.835 (30,4%) crianças recebeu o diagnóstico precoce ( Tabela 1 ). Não houve designação do subtipo no sistema de informação em 56,4% dos diagnósticos, que foram identificados apenas como TGD. Em seguida, os diagnósticos mais frequentes foram autismo infantil (31,9%) e TGD não especificado (7,0%). Apenas a variável sexo não apresentou diferença estatisticamente significativa (p = 0,502). A análise da variável raça/cor da pele foi inviável devido ao alto percentual de incompletude (39,3%), porém a análise restrita às categorias branca, preta, parda e amarela apresentou diferença estatisticamente significativa. O ano de 2013 apresentou maior proporção de diagnósticos do período (18,9%); mas de 2014 a 2018 a proporção aumentou progressivamente, com redução em 2019. Em relação ao diagnóstico precoce, variou de 23,3% em 2013 a 32,8% em 2019, com ápice em 2017 (33,4%) e 2018 (34,9%), com tendência linear estatisticamente significativa (p < 0,001).

**Tabela 1:** Distribuição proporcional (%) das características das crianças com diagnóstico de autismo e outros tipos de TGD atendidas no CAPSi segundo diagnóstico precoce ou tardio. Brasil, 2013 a 2019.

Características	Total		Diagnóstico				

			Precoce		Tardio		p^b^
		
	n	%	n	%	n	%	
Sexo							
Feminino	4075	18,1	1.221	17,9	2.854	18,2	0,502
Masculino	18.408	81,9	5.614	82,1	12.794	81,8	
Raça/cor							
Branca	5.835	26,0	1.880	27,5	3.955	25,3	< 0,001
Preta	461	2,1	107	1,6	354	2,3	
Parda	6.638	29,5	2.032	29,7	4.606	29,4	
Amarela	710	3,2	111	1,6	599	3,8	
Indígena	5	0,0	2	0,0	3	0,0	-
Ignorado	8.834	39,3	2.703	39,5	6.131	39,2	-
Origem							
Demanda espontânea	11.468	51,0	3.483	51,0	7.985	51,0	< 0,001
Atenção básica	9.719	43,2	3.081	45,1	6.638	42,4	
Outros^a^	1.296	5,8	271	4,0	1.025	6,6	
Região de residência							
Norte	1.358	6,0	248	3,6	1.110	7,1	< 0,001
Nordeste	5.495	24,4	1.867	27,3	3.628	23,2	
Centro-Oeste	897	4,0	249	3,6	648	4,1	
Sudeste	12.969	57,7	4.053	59,3	8.916	57,0	
Sul	1.764	7,8	418	6,1	1.346	8,6	
Município de residência igual ao de diagnóstico							
Sim	21.773	96,8	6.656	97,4	15.117	96,6	0,002
Não	710	3,2	179	2,6	531	3,4	
Diagnóstico inicial (CID)							
TGD geral (F84)	12.670	56,4	3.978	58,2	8.692	55,5	< 0,001
Autismo infantil (F84.0)	7.177	31,9	2.143	31,4	5.034	32,2	
Autismo atípico (F84.1)	628	2,8	127	1,9	501	3,2	
Outros TGD (F84.8)	445	2,0	133	1,9	312	2,0	
TGD não especificado (F84.9)	1.563	7,0	454	6,6	1.109	7,1	
Ano do diagnóstico							
2013	4.242	18,9	989	14,5	3.253	20,8	< 0,001
2014	2.490	11,1	723	10,6	1.767	11,3	
2015	2.669	11,9	782	11,4	1.887	12,1	
2016	2.932	13,0	914	13,4	2.018	12,9	
2017	3.529	15,7	1.179	17,2	2.350	15,0	
2018	3.645	16,2	1.271	18,6	2.374	15,2	
2019	2.976	13,2	977	14,3	1.999	12,8	
IDHM							
Muito baixo	761	3,4	232	3,4	529	3,4	0,008
Baixo	4.378	19,5	1.424	20,8	2.954	18,9	
Moderado	9.923	44,1	2.970	43,5	6.953	44,4	
Alto	7.421	33,0	2.209	32,3	5.212	33,3	

^a^Outros: serviço de urgência, outros Centros de Atenção Psicossocial, hospital dia e hospital psiquiátrico.

^b^Valor de p do teste qui-quadrado de Pearson.

Dentre os diagnósticos abordados, o Brasil apresentou, nesse período, uma proporção de diagnóstico de autismo infantil de 31,9% no CAPSi, variando de 23,7% no Norte a 39,0% no Nordeste. O TGD sem tipificação apresentou maior proporção no Norte (73,1%) e menor no Sul (36,5%), região que teve, porém, a maior proporção de TGD não especificado (17,5%). A idade média e mediana foi maior para autismo atípico, enquanto a mediana de idade foi a mesma para os demais transtornos. Em relação às regiões, observaram-se algumas variações: a região Norte apresentou maior média de idade para autismo atípico e menor para outros TGD – porém com ocorrência de poucos casos, e primeiro quartil superior à distribuição no Brasil para todos os diagnósticos. Para as regiões Nordeste e Sudeste, assim como para o Brasil, com exceção do diagnóstico de autismo atípico, o diagnóstico precoce ocorreu em 25% dos casos ( Tabela 2 ).

**Tabela 2:** Distribuição proporcional do diagnóstico de autismo e outros tipos de TGD atendidos no CAPSi e respectiva média e mediana de idade de diagnóstico segundo região de residência. Brasil, 2013_2019

Região de residência	Diagnóstico	n	%	Idade da criança (em anos)						

				Média	Desvio padrão	Mediana	1º Quartil	3º Quartil	Min.	Max.
Norte	Autismo atípico	13	1,0	7,3	2,0	6,0	6,0	9,0	5	11
	Autismo infantil	322	23,7	6,3	2,7	6,0	4,0	8,0	1	12
	TGD geral	993	73,1	6,1	2,8	6,0	4,0	8,0	1	12
	Outros TGD	2	0,1	4,5	0,7	4,5	4,3	4,8	4	5
	TGD não especificado	28	2,1	5,8	2,7	5,5	3,8	7,0	2	12
	Subtotal	1.358	100,0	6,2	2,7	6,0	4,0	8,0	1	12
Nordeste	Autismo atípico	203	3,7	5,9	2,8	5,0	4,0	7,5	1	12
	Autismo infantil	2.144	39,0	5,0	2,7	4,0	3,0	7,0	1	12
	TGD geral	2.798	50,9	5,2	2,8	5,0	3,0	7,0	1	12
	Outros TGD	133	2,4	5,3	2,9	5,0	3,0	7,0	1	12
	TGD não especificado	217	3,9	5,4	2,6	5,0	3,0	7,0	1	12
	Subtotal	5.495	100,0	5,2	2,8	4,0	3,0	7,0	1	12
Centro-Oeste	Autismo atípico	36	4,0	6,3	2,8	5,5	4,0	9,0	2	12
	Autismo infantil	235	26,2	5,6	2,8	5,0	3,0	8,0	1	12
	TGD geral	590	65,8	5,7	2,9	5,0	3,0	8,0	1	12
	Outros TGD	6	0,7	6,7	4,3	5,0	3,3	10,5	3	12
	TGD não especificado	30	3,3	5,9	2,9	6,0	4,0	8,0	1	12
	Subtotal	897	100,0	5,7	2,8	5,0	3,0	8,0	1	12
Sudeste	Autismo atípico	308	2,4	6,3	3,0	6,0	4,0	9,0	1	12
	Autismo infantil	3.815	29,4	5,5	2,8	5,0	3,0	7,0	1	12
	TGD geral	7.646	59,0	5,4	2,9	5,0	3,0	7,0	1	12
	Outros TGD	220	1,7	6,0	3,1	6,0	3,0	8,0	1	12
	TGD não especificado	980	7,6	5,6	2,9	5,0	3,0	7,0	1	12
	Subtotal	12.969	100,0	5,5	2,9	5,0	3,0	7,0	1	12
Sul	Autismo atípico	68	3,9	6,2	3,0	6,0	4,0	9,0	1	12
	Autismo infantil	661	37,5	6,3	2,9	6,0	4,0	8,0	1	12
	TGD geral	643	36,5	6,1	3,1	6,0	4,0	8,0	1	12
	Outros TGD	84	4,8	5,8	2,9	5,0	4,0	8,0	1	12
	TGD não especificado	308	17,5	5,6	3,1	5,0	3,0	8,0	1	12
	Subtotal	1.764	100,0	6,1	3,0	6,0	4,0	8,0	1	12
Brasil	Autismo atípico	628	2,8	6,2	2,9	6,0	4,0	9,0	1	12
	Autismo infantil	7.177	31,9	5,5	2,8	5,0	3,0	7,0	1	12
	TGD geral	12.670	56,4	5,4	2,9	5,0	3,0	7,0	1	12
	Outros TGD	445	2,0	5,8	3,0	5,0	3,0	8,0	1	12
	TGD não especificado	1.563	7,0	5,6	2,9	5,0	3,0	7,0	1	12
	Total	22.483	100,0	5,5	2,9	5,0	3,0	7,0	1	12

TGD: transtorno global do desenvolvimento.

A Tabela 3 apresenta os resultados do RR_b_e RR_a_, e os respectivos intervalos com 95% de confiança (IC95%). O diagnóstico precoce de crianças com autismo infantil foi 48% maior do que de autismo atípico. As demais categorias também apresentaram diagnóstico precoce maior que o autismo atípico: TGD sem designação de subtipo (RR_b_= 1,55), outros TGD (RR_b_= 1,48) e TGD não especificado (RR_b_= 1,44).

**Tabela 3:** Risco relativo bruto e ajustado dos fatores associados ao diagnóstico precoce das crianças com diagnóstico de autismo e outros tipos de TGD atendidas no CAPSi. Brasil, 2013 a 2019.

Variáveis	RRb	IC95%	RRa	IC95%
Diagnóstico inicial				
Autismo atípico	1,00		1,00	-
Autismo infantil	**1,48**	1,27–1,71	**1,43**	1,23–1,66
TGD geral	**1,55**	1,34–1,80	**1,50**	1,29–1,74
Outros TGD	**1,48**	1,21–1,81	**1,49**	1,21–1,83
TGD não especificado	**1,44**	1,22–1,69	**1,49**	1,26–1,76
Origem				
Outros^a^	1,00		1,00	
Atenção básica	**1,51**	1,37–1,68	**1,40**	1,26–1,55
Demanda espontânea	**1,45**	1,31–1,61	**1,29**	1,16–1,43
Ano do diagnóstico				
2013	1,00	-	1,00	-
2014	**1,25**	1,15–1,35	**1,25**	1,15–1,35
2015	**1,26**	1,16–1,36	**1,24**	1,15–1,35
2016	**1,34**	1,24–1,44	**1,33**	1,23–1,44
2017	**1,43**	1,34–1,54	**1,41**	1,37–1,58
2018	**1,50**	1,40–1,60	**1,47**	1,37–1,58
2019	**1,40**	1,31–1,52	**1,40**	1,30–1,50
Região de residência				
Norte	1,00	-	1,00	-
Nordeste	**1,86**	1,67–2,08	**1,90**	1,70–2,13
Centro-Oeste	**1,52**	1,31–1,76	**1,51**	1,31–1,76
Sudeste	**1,71**	1,54–1,90	**1,69**	1,52–1,88
Sul	**1,29**	1,14– 1,48	**1,27**	1,11– 1,45
Município de residência igual ao de diagnóstico				
Não	1,00	-	1,00	
Sim	**1,31**	1,10–1,55	**1,27**	1,11–1,42
IDHM^b^				
Muito baixo	1,00	-		
Baixo	1,07	0,95 - 1,20		
Moderado	0,98	0,88–1,10		
Alto	0,98	0,87–1,09		
Sexo				
Feminino	1,00	-		
Masculino	1,02	0,97–1,07		

^a^Outros: serviço de urgência, outros Centros de Atenção Psicossocial, hospital dia e hospital psiquiátrico.

^b^Valor de p na análise múltipla > 0,05.

As crianças encaminhadas pela atenção básica e aquelas cuja origem foi a demanda espontânea receberam mais diagnósticos precoces, respectivamente 51% e 45%, do que as oriundas de outros tipos de encaminhamento. Crianças que residiam no mesmo município em que receberam o diagnóstico apresentaram 31% mais diagnósticos precoces do que as demais. O diagnóstico precoce foi maior a partir de 2014 e menor na região Norte quando comparada às demais.

Na análise múltipla, a única variável que não foi incluída no modelo foi sexo, pois não apresentou significância estatística na análise bivariada (p > 0,20). A variável IDHM foi a única que não apresentou significância estatística na análise múltipla (p > 0,05) e, por isso, não foi mantida no modelo. Das variáveis incluídas no modelo para a análise múltipla, não houve mudança expressiva na magnitude da associação, ou seja, o efeito independente de cada variável após ser ajustado pelas demais foi similar ao não ajustado (bruto). O modelo apresentou, no entanto, baixa capacidade preditiva (2,0%) e um ajuste global inadequado (p < 0,001).

A Figura 2 apresenta a análise de resíduo que sinaliza ausência de casos influentes: a medida de alavancagem variou de > 0,000 a < 0,005, os resíduos padronizados de Pearson de -0,79 a 2,88, e nenhuma observação ficou dentro do ponto de corte da distância de Cook.

**Figura 2:** Resíduos padronizados de Pearson versus medida de alavancagem e respectiva distância de Cook do modelo de regressão para o diagnóstico precoce das crianças com diagnóstico de autismo e outros tipos de TGD atendidas no CAPSi. Brasil, 2013 a 2019.

## DISCUSSÃO

O estudo identificou um diagnóstico precoce e crescente ao longo dos anos estudados, com tendência linear estatisticamente significativa. A proporção de diagnósticos no período, no entanto, foi maior em 2013 e menor em 2019. Como em 2013 se iniciou o registro dos atendimentos realizados no CAPSi na RAAS^23^, é plausível supor que a mudança no instrumento de registro, anteriormente realizado em Autorização de Procedimentos de Alta Complexidade, possa ter gerado um acúmulo de registros, que foram lançados nesse primeiro ano. Já a redução em 2019 pode expressar uma queda real, necessitando de avaliação com uma série de anos maior.

Em estudo que avaliou 126 crianças autistas brasileiras residentes em 19 estados, a idade de diagnóstico variou de 16 a 204 meses, sendo que a maioria dos pais tinha curso superior (63,3%) e nível socioeconômico alto (76,4%)^24^. A idade de diagnóstico apresentou correlação fraca para idade do reconhecimento dos primeiros sinais do TEA e não apresentou correlação estatisticamente significativa com o nível socioeconômico da família, nem com a ordem de nascimento da criança e os sinais de autismo. Os autores sinalizaram, contudo, que é possível que em famílias com menor nível socioeconômico o diagnóstico de crianças com autismo ocorra mais tardiamente. Destacaram, ainda, que a média de idade de diagnóstico de autismo no Brasil é avançada quando comparadas a crianças diagnosticadas em outros países.

O diagnóstico tardio, entretanto, ainda é uma realidade internacional. Dentre as razões podem estar a falta de adesão de pediatras aos protocolos de avaliação, a variação de experiência e o uso de instrumentos não validados^25^. Em estudo de monitoramento realizado em 11 cidades dos Estados Unidos, a média de idade da criança na ocasião do diagnóstico foi de 51 meses, podendo ter variações em função do quociente de inteligência (QI) e da raça/cor da pele^26^. Já um estudo de revisão que avaliou a influência de características da criança, sua família e comunidade na idade de diagnóstico de TEA^27^encontrou variação média na idade de 38 a 120 meses e de 34 a 88 meses na idade mediana para todo o espectro e com tendência à redução ao longo do tempo. Os resultados indicaram, portanto, que o diagnóstico de crianças com autismo tem ocorrido cada vez mais cedo. Esse estudo também identificou que a gravidade do sintoma e o acesso aos sistemas de saúde e de educação foram fatores determinantes do diagnóstico precoce e que o desempenho das políticas e infraestrutura locais parecem causar diferenças na idade diagnóstica entre as regiões. Neste artigo, a idade média no Brasil, para o período estudado, variou de 5,4 anos (64,8 meses para TGD geral) a 6,2 anos (74,4 meses para autismo atípico), enquanto a mediana de idade foi de 5 anos (60 meses), exceto para o autismo atípico, que foi maior. Os critérios de exclusão de alguns diagnósticos de TGD utilizados sugerem que a idade média dos diagnósticos estudados, em crianças até 12 anos de idade, possa se aproximar da idade média diagnóstica do TEA no país, apesar de não terem sido mantidos os diagnósticos de outros transtornos desintegrativos da infância e Asperger. Além disso, variação da média de idade encontrada indica de fato um diagnóstico mais tardio no Brasil; e que ter limitado a população do estudo a crianças com até 12 anos de idade pode ter influenciado as médias encontradas, uma vez que o diagnóstico pode ocorrer em idades mais avançada.

Neste estudo a probabilidade de diagnóstico precoce diferiu entre as regiões, sendo maior para o Nordeste e o Sudeste quando comparados ao Norte, mesmo quando ajustada pelas demais variáveis incluídas no modelo. Diferenças entre as macrorregiões brasileiras na distribuição de diversos diagnósticos de atendimentos realizados nos CAPSi foram identificadas em estudo anterior^28^, o que pode afetar a ocorrência do autismo e de outros TGD. Ainda assim, estudo recente encontrou tendência crescente no diagnóstico precoce de crianças com TGD^16^. Embora a literatura aponte associação entre maior nível educacional dos pais e diagnóstico precoce^27^, a inclusão do IDHM, como um indicativo do nível educacional local, não se mostrou significativa. Os resultados deste estudo também mostram diferenças nas médias e medianas de idade ao diagnóstico entre as regiões, com médias maiores, no geral, para as regiões Norte e Sul, reiterando o diagnóstico mais tardio.

Realizar o diagnóstico no município de residência favorece a identificação precoce de TGD, mas a maioria dos municípios não dispõe de CAPSi^16^, embora se pressuponha que a rede de atenção à saúde seja regionalizada, com fluxos de atendimentos estabelecidos e a integralidade do cuidado garantida. O encaminhamento da atenção básica também contribuiu para o diagnóstico precoce, mas chama a atenção a alta associação com a demanda espontânea. Estudo recente abordou a relação entre a percepção de estigma por cuidadores de crianças com TEA e a dificuldade de acessar serviços de saúde na América Latina^29^. Um importante achado foi que as barreiras de acesso foram mais percebidas pelos cuidadores de crianças em idade pré-escolar e do sexo feminino. Especificamente em relação ao Brasil, os resultados indicaram que foram maiores as percepções de barreiras de acesso, a frustação em acessar o serviço, o sentimento de impotência e o impacto negativo percebido pelos cuidadores. Esses resultados destacam a dificuldade de acessar os serviços de saúde na fase em que deve ser realizado o diagnóstico precoce.

Verifica-se no país heterogeneidade na formação e distribuição dos profissionais e a disponibilidade de equipes multiprofissionais^2 , 30^, o que pode contribuir para a variabilidade dos diagnósticos de TGD entre as regiões, como identificado por Ceballos e colaboradores^28^e por este estudo. A maioria dos TGD foi não tipificado, porém na região Norte essa proporção foi superior a 73%, enquanto o autismo atípico representou 1%, patamares bem diferentes do valor nacional. Além disso, o autismo apresenta uma diversidade de sintomas, incluindo comorbidades associadas, e não conta ainda com determinantes biológicos ou ambientais estabelecidos que estejam associados à manifestação do transtorno^2^, sendo necessário, portanto, melhorar as rotinas diagnósticas, aprimorar e qualificar diretrizes para essas práticas, bem como para sistemas de apoio e de intervenção para a criança e seus familiares.

Podem contribuir para a compreensão dos fatores associados à ocorrência do TEA pesquisas que incluam variáveis contextuais, que, contudo, ainda são escassos. Neste estudo não foi possível utilizar a variável raça/cor da pele devido à incompletude dos registros e ao fato de a base de dados não incluir variáveis que permitissem uma avaliação mais abrangente, limitando a análise ao número restrito de variáveis disponíveis no sistema, o que provavelmente comprometeu a capacidade preditiva. Uma limitação do estudo é a ausência de informação sobre os métodos diagnósticos utilizados, o que poderia qualificar mais a discussão. O enriquecimento da base de dados por meio da inclusão de outras variáveis explicativas pode ajudar no ajuste do modelo. Ainda assim, este estudo é inédito ao usar dados públicos do RAAS para avaliar variáveis que influenciam o diagnóstico precoce.

## CONCLUSÃO

O diagnóstico precoce de autismo e outros TGD tem melhorado no país, mas ainda representa cerca de 30% dos diagnósticos realizados, sendo a maioria ainda não tipificado, o que pode comprometer a qualidade da assistência. O tipo de diagnóstico, a origem do encaminhamento, o local de residência e o ano de diagnóstico influenciaram sua ocorrência. As variáveis incluídas no modelo, embora tenham sido significativas, explicam pouco do diagnóstico precoce. Avançar em estudos que incluam variáveis ambientais e contextuais pode ajudar a ampliar a compreensão sobre o tema e contribuir para minimizar o impacto das oportunidades perdidas de intervenção, com consequente melhoria na qualidade de vida dessa população.
